# Sucking behaviour using feeding teats with and without an anticolic system: a randomized controlled clinical trial

**DOI:** 10.1186/s12887-018-1092-0

**Published:** 2018-03-16

**Authors:** Marina Kreitschmann, Lea C. Epping, Ariane Hohoff, Cristina Sauerland, Thomas Stamm

**Affiliations:** 1 Private Practice Dr. Thomas Hinz, Dr. Uta Neumann, Stoeckstraße 106, 44649 Herne, Germany; 2 Private Practice, Weststraße 1, 49176 Hilter, Germany; 3 Department of Orthodontics, University of Münster, Albert-Schweitzer-Campus 1, 48149 Münster, Germany; 4 Institute of Biostatistics and Clinical Research, University of Münster, Schmeddingstraße 56, 48149 Münster, Germany

**Keywords:** Vented teat, Bottle-feeding, Infants, Infantile colic, Feeding-teat, Aerophagia

## Abstract

**Background:**

This study aimed to investigate differences in sucking behavior of infants bottle-fed with vented (so-called anticolic) teats (VTs) and nonvented teats (NVTs).

**Methods:**

Trial design: Prospective, randomized clinical trial. Ninety-six term, healthy infants (aged 1–8 months) were assessed for eligibility. Seventy-three infants remained for intention-to-treat (ITT) and 65 infants (vented group: *n* = 31; nonvented group: *n* = 34) for the per-protocol (PP) analysis. During bottle-feeding, sucks/min, pauses/min, amount of formula intake (mL), feeding time (min), heart rate (bpm), respiratory rate (bpm), and oxygen saturation (%) were recorded. In addition, a parental survey was carried out to reveal possible symptoms of infantile colic. Sample-size calculation and confirmatory and exploratory analyses were performed using the Mann-Whitney *U* test and Fisher’s exact test.

**Results:**

Except for the parameter sucking pauses per minute (NVTs > VTs, *p* = .03), no differences between groups were found with the ITT and PP analysis. After excluding infants with a disproportionately complementary diet (subgroup analysis, infants aged 1–6 months, *n* = 54) the primary outcome (sucks per minute) showed significant differences (NVTs > VTs, *p* = .01). The amount of formula intake, feeding time, and cardiorespiratory parameters were similar in both groups. The parental survey did not show any relation between types of feeding teats and possible symptoms of infantile colic.

**Conclusions:**

Compared with an NVT group, infants aged 1–6 months need fewer sucks and pauses when fed with VTs. In both groups, equal amounts of feeding medium and feeding time were observed. With NVT feeding, disruption occurs when the bottle vacuum is released by air from the oral cavity. Therefore, higher sucking frequency is needed to rebuild the oral vacuum for bottle milk flow, which implies higher risk of aerophagia. Overall, we suggest that the VTs provided a more coordinated drinking pattern than did the NVTs, which may have a positive effect on gastric distress.

**Trial registration:**

Trial Registration: DRKS-Trial Registration No. DRKS00004885. Registered April 16, 2013. Universal Trial No. U1111–1141-5857.

## Background

Infantile colic portrays a widespread problem with an uncertain prevalence of 5%–40% [1] within the first 4 [2] or 6 months [1] of an infant’s life. The occurrence is difficult to identify due to differences in classification, methods of data collection, study design, and parents’ perception of defining colic [1].

Although the history of research now reaches over 115 years, based on the paper of Zahorsky [3], the etiology of infantile colic still remains unknown [4]. As a result, therapeutic interventions to reduce the severity of symptoms and crying episodes are lacking their effectiveness [4, 5] and stressed parents seeking alternative methods to cope with their suffering infants. In this situation parents are susceptible to promises made by manufacturers of feeding bottles. Numerous bottle-nipple systems (BNSs) are available on the market, advertised to reduce infantile colic. The idea behind those so-called “anticolic” teats is to prevent excessive air swallowing (aerophagia) during feeding. It is estimated that 70% of the gastrointestinal gas is swallowed [6] and it was hypothesized that a substantial proportion of air could accumulate, leading to symptoms of distension, discomfort [7, 8], or colic [9, 10].

Studies on the relationship between vented BNS and reduction of infant colic symptoms are limited. Available information is based on subjective assessments like expert opinion [11], parents’ recordings of infant’s level of arousal, sleep states [12] and questionnaires to rank infant’s symptoms on a Likert-type scale [13].

Studies on direct measurement of air swallowing during bottle-feeding are not available. However, BNSs were assessed concerning suck-swallow-breath coordination in relation to breastfeeding [7, 14]. It was speculated that increased air swallowing leads to air accumulation in the stomach which may cause gastric upset and that pulse oximetry measures may help to clarify post feeding distress [7].

To examine the effect of a vented “anticolic” teat on suck-swallow-breath coordination we investigated the sucking behaviour of infants bottle-fed with vented teats (VTs) and nonvented teats (NVTs). We hypothesize that an uncoordinated random-like sucking behaviour implies more stress in terms of increased sucking frequency, oxygen desaturation, increased cardiorespiratory parameters, leading to a higher risk of aerophagia.

## Methods

### Trial design

**Fig. 1:**
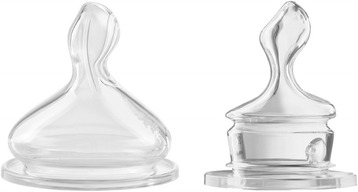
Used feeding teats. Left: Vented teat Nuk First Choice Plus. Right: Nonvented teat Nuk Classic. Both, Nuk, Mapa, Zeven, Germany

The present study was a randomized controlled clinical trial conducted from November 2013 to July 2015 in Muenster (North-Rhine-Westphalia, Germany) and Berlin, Germany. We investigated two different feeding teats (Nuk First Choice Plus and Nuk Classic, Mapa, Zeven, Germany), one of which was specifically developed (according to the manufacturer) to prevent infantile colic. It has a device (an “anticolic valve”) at the base of the teat through which air can pass into the bottle during drinking, thus preventing vacuum formation. The other feeding teat has no anticolic system, serving for the control group (Fig. 1). Both teats have a so-called orthodontic shape. Cardiorespiratory parameters during feeding were recorded by an ECG monitor.

### Changes to trial design

Recording of the cardiorespiratory parameters—heart and respiratory rates and oxygen saturation—was changed from once during the drinking process to 5 min prior to the feeding procedure and 10 min after feeding to consider potential differences in the initial situation of the infants. Due to a disappointing recruitment rate in Muenster, we finally had to choose an additional location for recruitment, namely, the Department of Orthodontics in Berlin.

### Participants

Eligibility criteria were as follows: (i) Caucasian neonates whose mothers delivered in the 38th week of gestation or later, (ii) healthy neonates, (iii) neonates whose parents decided in advance to feed by bottle exclusively or whose breast-feeding had terminated at least 8 weeks prior, (iv) postnatal age of 1–8 months, (v) dietary supplement was allowed, (vi) medication was permitted, but had to be noted precisely by the parents.

Exclusion criteria were as follows: (i) upper respiratory infection (ii) anomalies of the oro-facial region (iii) known suckling or swallowing disorders, (iv) already known intolerances to food components, (v) twins or other multiples.

Eligibility determination as well as the measurements took place at the orthodontic departments of the University Clinic of Muenster and Charité, University Clinic of Berlin.

### Interventions

Written informed consent was obtained from both parents of each infant who participated in the study. For the purpose of the study, parents received randomly allocated feeding teats with corresponding bottles, and the infants were given 2–3 weeks of acclimatization during which they were to be fed exclusively by the feeding teats received prior to the appointment for measurement. Randomization was stratified by gender and a random integer list of 0 and 1 (random.org). Parents were instructed to complete a self-administered, non-validated questionnaire (Table 1) after 1 week of the acclimatization phase to reveal possible symptoms of infantile colic.

**Table 1:** Questionnaire Items Group B - vented teat and nonvented teat groups

Item	Nonvented Teat Group (*n* = 29)	Vented Teat Group (*n* = 25)
1. My/our infant chokes while drinking (*n*).		
Never	6	3
Rarely	20	18
Always	3	4
2. My/our child spits out a significant amount of milk after drinking.		
Never	4	5
Rarely	18	15
Always	7	5
3. My/our child cries at least 3 days per week and 3 h or more per day.		
Yes	1	2
No	28	23
4.The intervals in which the child cries or screams begin abruptly.		
Yes	6	3
No	23	22
5. My/our child has a bloated, hard stomach after feeding.		
Never/rarely	24	15
Occasionally/often	5	10
6. I/we notice increased muscle tension, clenched fists, and drawn-up legs against the child’s abdomen.		
Never/occasionally	24	22
Often	4	3
No information	1	0
7. I/we notice flatulence in our child.		
Never	7	2
Rarely	8	10
Occasionally/often	14	13
8. During the phases of excessive crying, the child’s cries are more piercing, brighter, or shriller than usual.		
Yes	5	4
No	24	21
9. My/our child is inconsolable during the phases of excessive crying and cannot be calmed.		
Yes	3	3
No	26	22
10. The phases during which the child cries excessively and is difficult or impossible to soothe are timed.		
Throughout the day	7	2
Especially in the late afternoon and evening	1	2
Especially in the evening and at night	2	4
At other times	3	3
No information /no evaluation	16	14
11. Our child was administered the following medications during the study phase (please note all medications, even nonprescription).		
No evaluation	3	3
None	7	9
Others	16	9
Antibiotics	1	1
Gastrointestinal therapeutics (Sab Simplex, Lefax)	2	3
12. If a complementary diet was given, please state exactly what was given and at what time.		
No evaluation	1	2
Yes	13	7
No	15	16
13. We experienced problems with the feeding teat.		
Yes	13	6
No	16	19

**Fig. 2:**
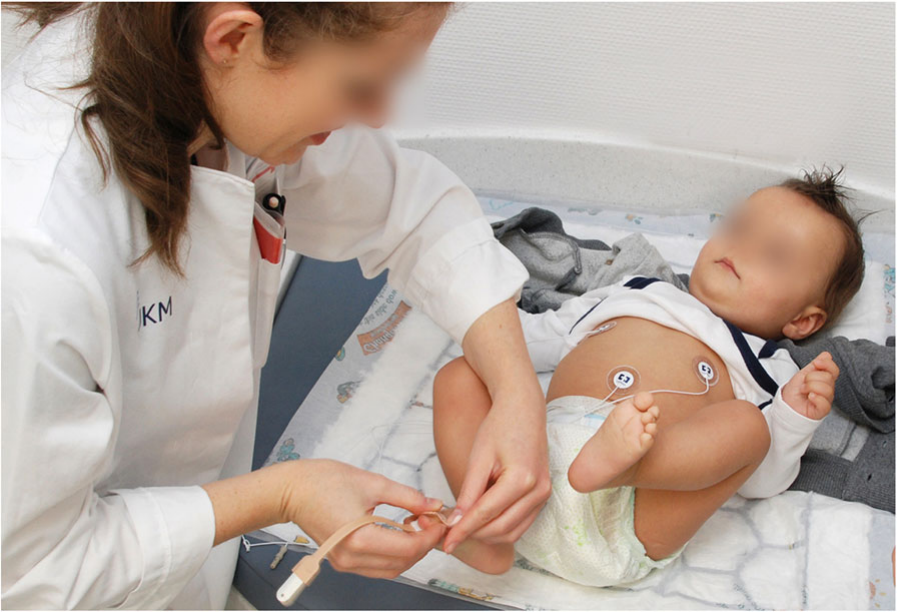
Electrodes placed on the infant and connected to the ECG monitor according to the manufacturer’s information

**Fig. 3:**
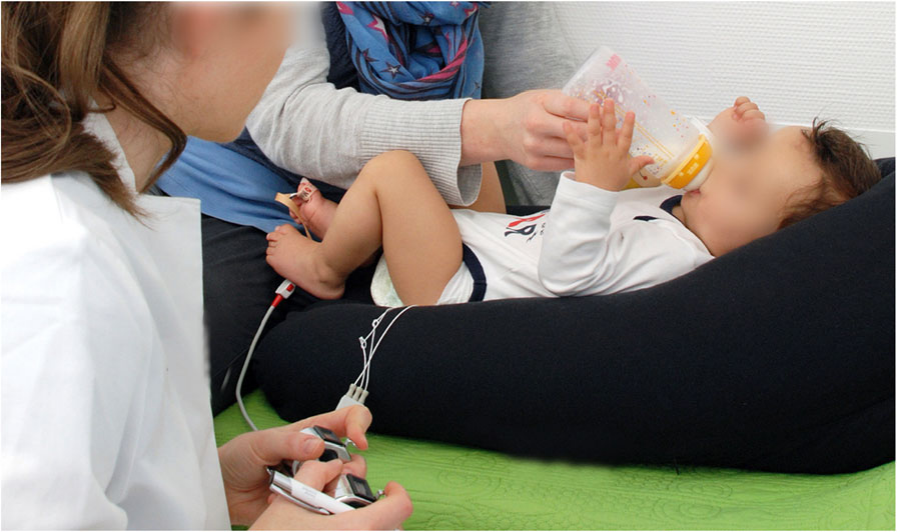
Examiner records sucks and pauses by direct observation

Following acclimatization, the parents made a one-time appointment at one of the clinics mentioned above. Here, the children were connected to an ECG monitor (Vitaguard VG 3100, Getemed Medizin und Informationstechnik AG, Teltow, Germany), which recorded their heart and respiratory rates and oxygen saturation (Fig. 2). The recording and feeding were done in a quiet, closed room to minimize disturbances. Infants were fed in a supine, semi-upright position by their parents (Fig. 3).

Two examiners, not blinded to the study, were involved to take all records. Both defined and agreed on the characteristics what constitutes sucking and swallowing before the study. Since the lifting of the larynx was difficult to detect (the chin of the child laid on the chest during drinking) sucks were defined as the rhythmic forward and backward motion of the lower jaw [15]. Interruption of this rhythmic movement was defined as a pause.

**Fig. 4:**
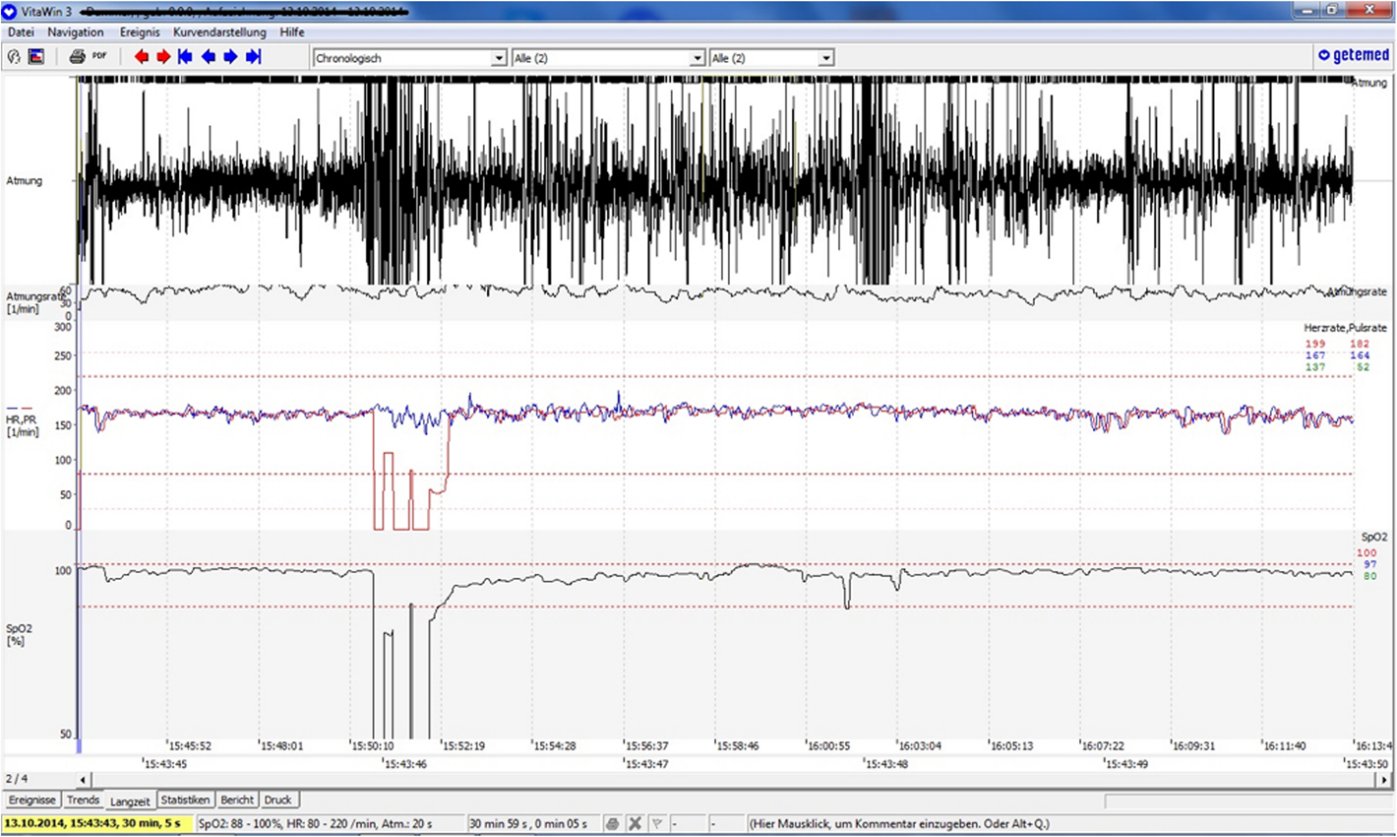
Graphical representation of heart and respiratory rates and oxygen saturation with VitaWin 3 (Getemed Medizin-und Informationstechnik AG, Teltow, Germany)

During each study session one examiner took the records three times: (t1) 5 min before feeding, (t2) during feeding with parallel observation and documentation of sucking and swallowing patterns, and (t3) 10 min after feeding (Fig. 4). During the feeding procedure, the children themselves determined the time and amount of feeding until the infant had stopped drinking by himself. Following that, results from observation of the drinking patterns the cardiorespiratory parameters and the information from the parents’ questionnaire were examined for possible associations.

### Statistical methods

Statistical analyses were performed using SAS software, version 9.4 of the SAS System for Windows (SAS Institute, Cary, NC) and IBM SPSS Statistics 23 for Windows (IBM Corp, Somers, NY).

According to the intervention’s objectives, the primary outcome of the trial was the number of sucks/min while pauses/min, feeding time, heart rate, respiratory rate, oxygen saturation, volume of milk intake, and data from the questionnaire were secondary outcomes.

Sample size calculation was performed under the assumption of a mean number of 70 sucks/min and a standard deviation of 9 sucks/min [16]. Differences in the primary outcome variable (sucking frequency) were considered relevant if they were in the order of a magnitude of at least 10%. Based on this information and a significance level of 5%, the necessary sample size comprised 29 evaluable cases per group to detect relevant differences in the two-sided Mann-Whitney *U* test with 80% statistical power.

The data were described for categorical variables by absolute and relative frequencies and for continuous variables by mean, standard deviation, median, and range. Categorical variables were compared between groups by Fisher’s exact test and for continuous variables using the Mann-Whitney *U* test. *P* values <.05 were considered to be statistically significant. All *p* values reported were two-sided.

## Results

### Subjects

**Fig. 5:**
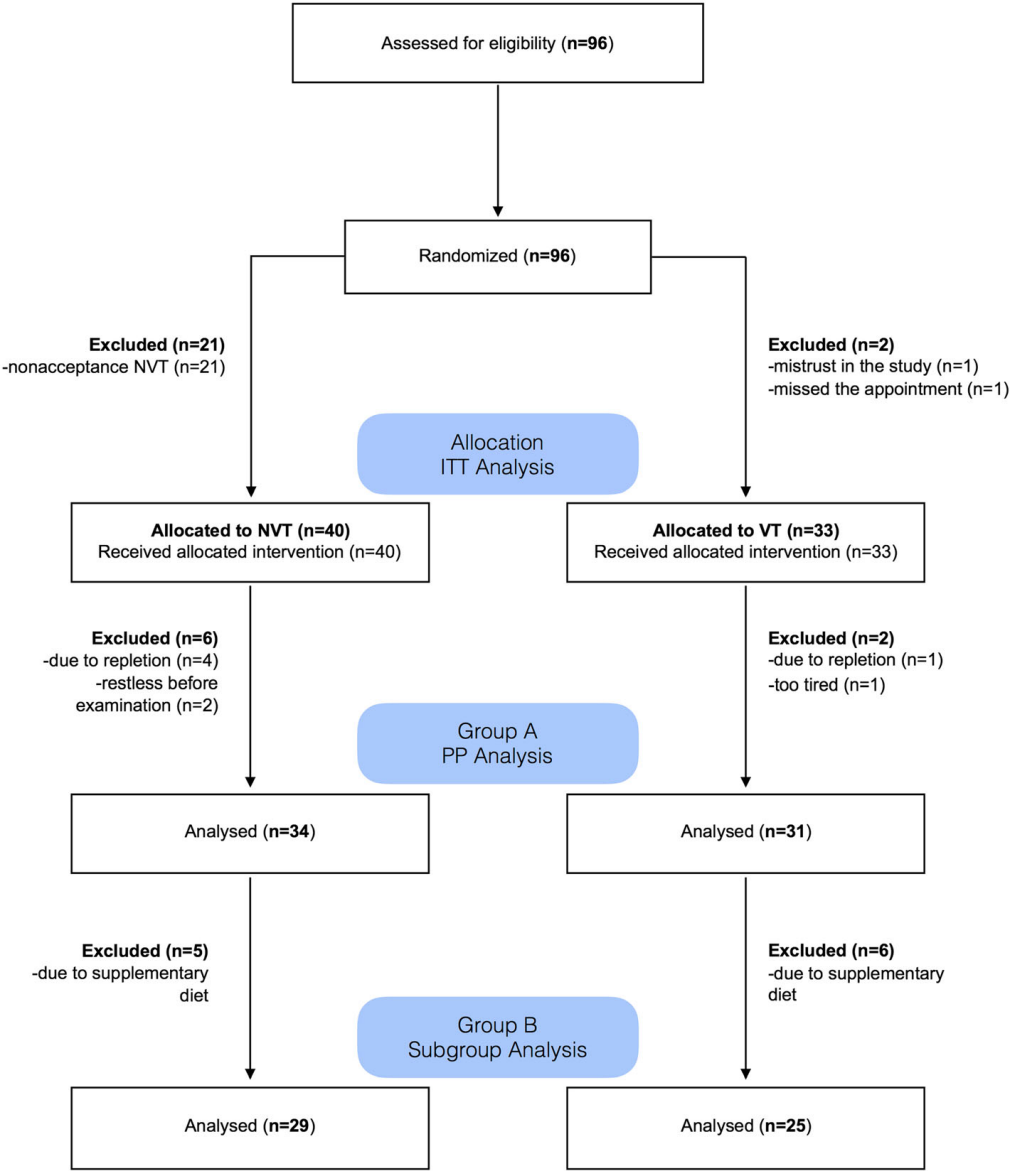
Flow diagram according to the CONSORT statement

Of a total of 96 enrolled infants, 21 interrupted their contributions due to nonacceptance of the conventional NVT. One participant with a VT discontinued because of mistrust in the study and another missed the agreed appointment (Fig. 5).

Seventy-three infants remained for the intention-to-treat (ITT) analysis (NVT, *n* = 40; VT, *n* = 33). During the course of the study, a total of eight children (NVT, *n* = 6; VT, *n* = 2) were excluded because they did not want to drink or were restless, tired, or saturated; so, 65 infants remained for the per-protocol (PP) analysis.

Analysis of the questionnaire revealed a significant relationship between infant age (> 6 months) and the complementary diet (*p* < .0001). We therefore excluded infants older than 6 months for a subgroup analysis to assess the effect of a complementary diet.

### Measurements

The ITT (Table 2) and PP analysis revealed no differences between the groups except the parameter “sucking pauses per minute”. There was no difference in drinking time (*p* = .13, *p* = .10) and the amount of formula intake (*p* = .15, *p* = .20), but infants fed with nonvented teats needed more pauses (*p* = .03, *p* = .02) than did infants fed with vented teats. Neither gender nor age had an influence on the measurements obtained.

**Table 2:** Main group (intention-to-treat analysis). Baseline characteristics by vented teat group and nonvented teat group

	Total	Vented Teat Group	Nonvented Teat Group	*P* Value
Main Group, n (%)	73 (100)	33 (45)	40 (55)	
Age, months				0.72
Mean (SD)	4.6 (2.1)	4.5 (2.2)	4.7 (21.1)	
Median (Range)	5	4 (1–8)	5 (0–8)	
Gender, n (%)				0.64
Male	38 (52)	16 (48.5)	22 (55)	
Female	35 (48)	17 (51.5)	18 (45)	
Characteristics during bottle-feeding				
Sucks/min				0.63
Mean (SD)	40.0 (17.8)	38.7 (16.8)	41.1 (18.7)	
Median (Range)	38.2 (9.7–78.6)	36.3 (12.7–78.6)	40.7 (9.7–70.1)	
Pauses/ min				0.03
Mean (SD)	2.5 (1.3)	2.1 (1.3)	2.7 (1.2)	
Median (Range)	2.0 (0.0–6.0)	2.0 (0.0–6.0)	3 (0.5–6.0)	
Amount of formula intake (ml)				0.15
Mean (SD)	136.1 (57.2)	147.6 (58.2)	126.7 (55.4)	
Median (Range)	120.0 (25.0–250.0)	140.0 (50.0–250.0)	120 (25.0–230.0)	
Feeding time (minutes)				0.13
Mean (SD)	12.1 (6.5)	10.4 (3.7)	13.5 (7.8)	
Median (Range)	11.0 (3.5–37.0)	10.0 (5.5–19.6)	11.4 (3.5–37.0)	
Vital parameters				
Heart rate (bpm)				
Before feeding Median (Range)	143.0 (94.0–201.0)	141.0 (115.0–199.0)	144.0 (94.0–201.0)	0.74
During feeding Median (Range)	153.0 (130.0–184.0)	153.0 (139.0–178.0)	154.0 (130.0–184.0)	0.57
After feeding Median (Range)	148.0 (123.0–181.0)	146.0 (125.0–176.0)	148.0 (123.0–181.0)	0.77
Respiratory rate (bpm)				
Before feeding Median (Range)	41.0 (30.0–59.0)	40.0 (31.0–59.0)	41.0 (30.0–54.0)	0.35
During feeding Median (Range)	45.0 (36.4–61.0)	44.0 (38.0–61.0)	46.0 (36.4–60.0)	0.43
After feeding Median (Range)	41.0 (30.0–58.0)	39.0 (33.0–58.0)	41.5 (30.0–50.0)	0.52
Oxygen Saturation (%)				
Before feeding Median (Range)	98.0 (87.0–100.0)	99.0 (93.0–100.0)	98.0 (87.0–100.0)	0.01
During feeding Median (Range)	98.0 (88.0–100.0)	98.0 (88.0–100.0)	98.0 (90.0–100.0)	0.71
After feeding Median (Range)	98.0 (84.0–99.0)	98.0 (84.0–99.0)	98.0 (90.0–99.0)	0.82

*Abbreviation*: *ml* millilitre, *bpm* beats per minute, *bpm* breaths per minute

**Fig. 6:**
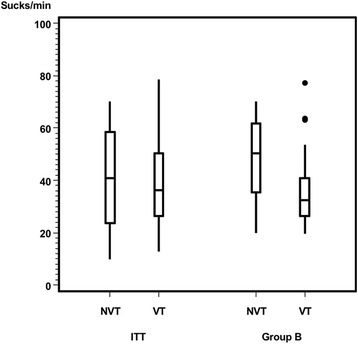
Primary outcome sucks per minute between NVTs (ITT: 41.1 ± 18.7; Group B: 48.4 ± 15.6) and VTs (ITT: 38.7 ± 16.8; Group B: 36.7 ± 15.2). ITT (*p* = .63), Group B (*p* = .01)

**Fig. 7:**
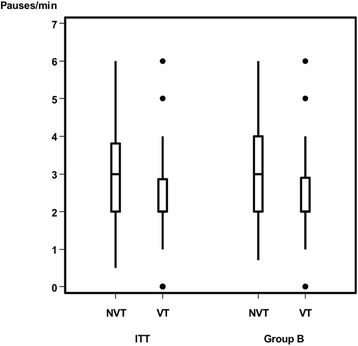
Pauses per minute between NVTs (ITT: 2.7 ± 1.2; Group B: 2.9 ± 1.3) and VTs (ITT: 2.1 ± 1.3; Group B: 2.3 ± 1.3). ITT (*p* = .03), Group B (*p* = .06)

**Fig. 8:**
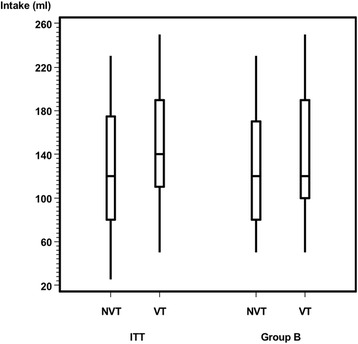
Primary outcome formula intake (mL) between NVTs (ITT: 126.7 ± 55.4; Group B: 127.8 ± 52.2) and VTs (ITT: 147.6 ± 58.2; Group B: 143.4 ± 56.4). ITT (*p* = .15), Group B (*p* = .33)

**Fig. 9:**
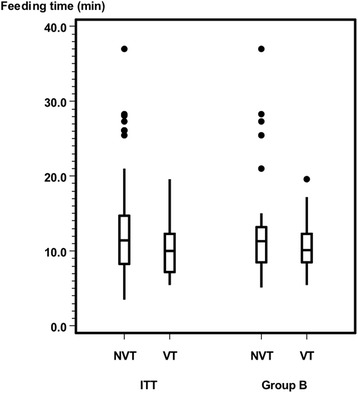
Feeding time in minutes for the NVT (ITT: 13.5 ± 7.8; Group B: 13.2 ± 7.5) and VT group (ITT: 10.4 ± 3.7; Group B: 10.6 ± 3.5). ITT (*p* = .13), Group B (*p* = .34)

After excluding infants with a disproportionately complementary diet (subgroup B analysis, Table 3) the primary outcome (sucks/min) showed significant differences (*p* = .01) between the VT and NVT group (Fig. 6). The VT group showed significantly fewer pauses per minute than did the NVT group in the ITT and PP analysis, which is a trend (*p* = .06) only in the subgroup B analysis (Fig. 7). In Group B, 65.5% (19 / 29) of the infants with nonvented teats had ≤3 pauses/min. In contrast, this proportion was 88% (22/25) for infants with vented teats. Both the amount of formula intake (Fig. 8) and feeding time (Fig. 9) were similar in both groups.

**Table 3:** Subgroup B analysis. Baseline characteristics by vented teat group and nonvented teat group

	Total	Vented Teat Group	Nonvented Teat Group	*P* Value
Subgroup, n (%)	54/73 (74)	25/33 (76)	29/40 (73)	
Age, months				0.52
Mean (SD)		3.76 (1.64)	4.03 (1.68)	
Median (Range)		4 (1–6)	4 (1–6)	
Gender, n				1.00
Male	27	12	15	
Female	27	13	14	
Chracteristics during bottle-feeding				
Sucks/min				0.01
Mean (SD)	43.0 (16.4)	36.7 (15.2)	48.4 (15.6)	
Median (Range)	39.0 (19.7–77.1)	32.4 (19.7–77.1)	50.4 (19.8–70.1)	
Pauses/ min				0.06
Mean (SD)	2.7 (1.3)	2.3 (1.3)	2.9 (1.3)	
Median (Range)	2.3 (0.0–6.0)	2.0 (0.0–6.0)	3.0 (0.7–6.0)	
Amount of formula intake (ml)				0.33
Mean (SD)	135.0 (54.2)	143.4 (56.4)	127.8 (52.2)	
Median (Range)	120.0 (50.0–250.0)	120.0 (50.0–250.0)	120.0 (50.0–230.0)	
Feeding time (minutes)				0.34
Mean (SD)	12.0 (6.1)	10.6 (3.5)	13.2 (7.5)	
Median (Range)	10.8 (5.2–37.0)	10.2 (5.5–19.6)	11.3 (5.2–37.0)	
Vital parameters				
Heart rate (bpm)				
Before feeding Median (Range)	144.5 (107.0–201.0)	142.0 (123.0–199.0)	147.0 (107.0–201.0)	0.33
During feeding Median (Range)	156.5 (130.0–183.0)	153.0 (140.0–177.0)	159.0 (130.0–183.0)	0.68
After feeding Median (Range)	149.5 (124.0–177.0)	146.0 (131.0–176.0)	153.0 (124.0–177.0)	0.34
Respiratory rate (bpm)				
Before feeding Median (Range)	41.5 (31.0–59.0)	41.0 (31.0–59.0)	42.0 (35.0–54.0)	0.16
During feeding Median (Range)	45.0 (38.0–61.0)	44.0 (38.0–61.0)	46.9 (38.0–60.0)	0.21
After feeding Median (Range)	42.7 (31.0–58.0)	42.0 (33.0–58.0)	43.0 (31.0–50.0)	0.6
Oxygen Saturation (%)				
Before feeding Median (Range)	99.0 (87.0–100.0)	99.0 (93.0–100.0)	98.0 (87.0–100.0)	0.16
During feeding Median (Range)	98.0 (88.0–100.0)	98.0 (88.0–100.0)	98.0 (90.0–100.0)	0.97
After feeding Median (Range)	98.0 (90.0–99.0)	98.0 (91.0–99.0)	98.0 (90.0–99.0)	0.83

Abbreviation: *ml* millilitre, *bpm* beats per minute, *bpm* breaths per minute

**Fig. 10:**
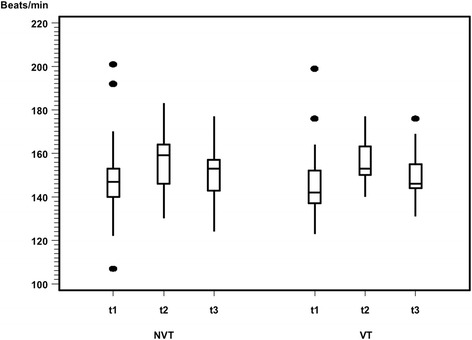
Heart rates in beats per minute (bpm) before, during, and after feeding

Heart rates were within normal limits and showed a similar pattern in both groups (Fig. 10). Heart rates increased by 8.9 ± 10.9 bpm during feeding (from t1 to t2) and decreased by 6.1 ± 7.4 bpm after feeding (from t2 to t3). The VT group showed consistently lower median bpm values than did the NVT group at each recording time, but not to a significant extent.

**Fig. 11:**
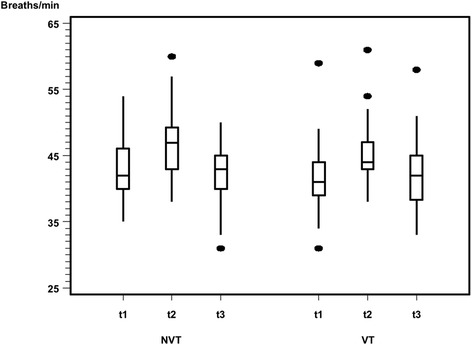
Respiratory rate in breaths per minute (bpm) before, during, and after feeding. The VT group showed consistently slightly lower median bpm values than did the NVT group at each recording time, but not to a significant extent

Respiratory rate had similar characteristics. On average, the rate increased by 3.9 ± 4.8 bpm during feeding (from t1 to t2) and decreased by 4.2 ± 5.2 bpm after feeding (from t2 to t3). Again, with respect to recording times, the VT group showed consistently lower median breaths/min than did the NVT group but also not to a significant extent (Fig. 11).

Oxygen saturation was consistently in the normal range, at approximately 97% at any recording time. There was no difference between the groups.

### Questionnaire

The questionnaire by itself did not show any differences in the ITT, PP, or subgroup B analysis between both types of feeding teats regarding possible symptoms of infantile colic (Table 1). There was no difference in any parameters in infants who took medication and those who did not.

## Discussion

The aim of this study was to investigate differences in sucking behaviour of infants bottle-fed with vented and nonvented teats. We hypothesized that possible differences of milk flow may result in uncoordinated sucking, implying more stress in terms of oxygen desaturation, increased heart and respiratory rates, and increased sucking frequency, leading to a higher risk of aerophagia. We used a mixed approach consisting of a parents’ self-administered, non-validated questionnaire and a monitoring of infants’ heart and respiratory rates and oxygen saturation before, during, and after feeding.

Various studies have investigated topics related to nutritive and nonnutritive sucking and their mechanisms and various BNSs and how they influenced the infant, but as far as we are aware, no previous studies have been published, comparing the sucking behaviour in full term infants using vented and nonvented teats. For this reason, our results cannot be discussed in view of comparable investigations.

One focus of research is the comparison of breast- and bottle-feeding. Despite high variability in breastfeeding studies, sucking behaviour improves with maturation [14] and bottle fed term infants show lower breathing frequency [17], lower oxygen saturation [7, 17], higher heart rate and lower blood pressure [18], lower suck frequency [15, 17], less coordinated (random) sucks [7], and less sucking pauses [15].

Nipple units differ in size, shape, consistency and mechanics and these factors are thought to influence suck-swallow-breath coordination in both term and preterm infants [7, 16, 19–21].

We found that sucking frequency using VTs was lower in the ITT analysis and significantly lower in the subgroup B analysis (*p* = .01). A comparative investigation of vented and nonvented bottles in preterm infants showed results nearly similar to ours [21]. The authors observed that sucking frequency is lower in a vacuum-free bottle system which confirmed “a more mature stage of sucking” [21]. The preterm infants showed a sucking frequency of 0.6 sucks/s with a vacuum-free bottle system and 0.9 sucks/s with a standard bottle, which corresponds to 36 sucks/min with a vacuum-free bottle system and 54 sucks/min using a standard bottle, closely matching our results (Table 3).

Moral and coworkers used the same VT as in our study when comparing breast- and bottle-feeding [15]. They found in a group of exclusively bottle-fed infants 37.9 ± 13.5 sucks/min which corresponds closely to our findings (Tables 2, 3). Infants 3–5 months of age showed significantly less pauses during bottle-feeding compared to breast-feeding [15]. In contrast, other studies found higher sucking values when different nonvented nipples were used [16].

Various studies focus on the influence of a specific bottle or nipple design on a particular health parameter of the infant. These studies comparing BNSs focused, inter alia, on vital parameters such as oxygen saturation during bottle-feeding with a particular feeding teat design [7, 20, 22] and sucking skills [7, 21].

Fucile et al. investigated skills of suck-swallow-respiration coordination and observed higher sucking stages when fed with the VT bottle [21]. This more mature sucking [21] corresponds to our own findings: We found no differences between the amount of formula intake and feeding time throughout the feeding procedure, meaning that, with the same amount of feeding medium for the same time, subgroup B needed fewer sucks and less pauses with the VTs than with the NVTs. Clinically, lower mean suck frequency suggests that the nipple enables to lengthen the intrasuck interval to allow the time necessary for swallowing larger volume of milk [22]. Our findings indicate that, on the one hand, the VTs did not hasten the formula flow nor did they increase formula intake. On the other hand, they did foster a more constant nonrandom drinking process.

Even though the drinking process is different between the VT and NVT group, we found no differences concerning cardiorespiratory measurements. Our results support the findings of Fadavi et al. who observed no differences in oxygen saturation when term neonates were bottle-fed with different nipples [22]. This is in contrast to other studies that found decreased oxygenation saturation during feeding of term neonates [7, 17]. One possible explanation for our results may be that we included older infants who maintained stable oxygen saturation.

Preterm infants have significant desaturation during bottle-feeding [23], but it could be shown that oxygen saturation increases significantly if a vented BNS is used [20]. Interestingly, some authors reported significantly lower SpO2 after feeding and attribute this to aerophagia in terms of burping and gastric distress [7, 24]. In general, higher oxygen levels during bottle-feeding is seen as a more coordinated sucking, swallowing, and breathing pattern [7]. The authors stated, “If a system can be designed that promotes less swallowing, babies can feed more like the natural physiologic norm of breast-feeding.” [7].

Results from the literature and our own findings suggest that nonvented teats have a higher risk for aerophagia. The mechanism behind nonvented teats is the vacuum that builds up within the bottle and results in a net decrease of milk flow [21]. The infant tries to compensate for the negative pressure by increasing sucking frequency or amplitude until nipple release after air reflux from the oral cavity. Vented teats allow the nipple to deliver formula in an uninterrupted process [20].

The hypothesis that aerophagia causes colic symptoms [3, 10, 25] is unproven, and the evidence of vented BNS on infant colic is very low. Subjective assessments like expert opinion [11] and questionnaires [12, 13] attribute a positive effect of vented BNS on infant colic. Other studies found that aerophagia could be seen as a consequence of increased sucking frequency, which may cause gastric upset [7, 24]. Our own results also support the findings that increased sucking implies the risk of aerophagia which could be reduced by using vented BNSs. In our investigation, the used questionnaire by itself did not show any differences between the two types of feeding teats in connection with colic symptoms. However, valid tools to assess infant colic are not available [26] and further studies are needed to prove the relation between aerophagia and infant colic.

The age of the infants is a confounding factor and a potential limitation of this study. Sucking behaviour improves with age and the inclusion of infants older than 6 months may have biased the sample. We included older infants due to a disappointing recruitment rate of exclusively bottle-fed healthy infants. Complementary diet increased with age and showed an effect on the ITT and PP analysis. Therefore, 6 to 8 months old infants were excluded.

Maturation may also be the reason why both groups maintain stable cardiorespiratory parameters and the effect of a ventilation is too small to create significant differences between the groups. Preterm or other impaired infants may be more vulnerable to this effect.

Specific bottle or nipple designs have an influence on sucking behaviour. We therefore used the same “orthodontic” shape of one manufacturer for both groups. The different base size may have an effect but lip resting was not disturbed with both teats.

## Conclusions

Our hypothesis that an uncoordinated sucking behaviour implies more stress in terms of increased sucking frequency could be confirmed, whereas the effect of oxygen desaturation and cardiorespiratory parameters must be rejected. Compared with an NVT group, infants aged 1–6 months need fewer sucks and pauses when fed with VTs. In both groups, equal amounts of feeding medium and feeding time was observed. With NVT feeding, disruption occurs when the bottle vacuum is released by air from the oral cavity. Therefore, higher sucking frequency is needed to rebuild the oral vacuum for bottle milk flow, which implies a higher risk of aerophagia. The role of aerophagia in the occurrence of infantile colic is vague and must be investigated further. Overall, we suggest that the VTs provided a more coordinated drinking pattern than did the NVTs, which may have a positive effect on gastric distress.

## Abbreviations

BNS: Bottle-nipple system
ITT: Intention- to- treat
NVT: Nonvented teat
PP: Per-protocol
VT: Vented teat
